# Adaptive Lévy Walks in Foraging Fallow Deer

**DOI:** 10.1371/journal.pone.0006587

**Published:** 2009-08-11

**Authors:** Stefano Focardi, Paolo Montanaro, Elena Pecchioli

**Affiliations:** 1 ISPRA, Sede amministrativa INFS, Ozzano Emilia, Italy; 2 IASMA Research and Innovation Centre, Fondazione Edmund Mach, Environment and Natural Resources Area, S. Michele all'Adige, Italy; University of Utah, United States of America

## Abstract

**Background:**

Lévy flights are random walks, the step lengths of which come from probability distributions with heavy power-law tails, such that clusters of short steps are connected by rare long steps. Lévy walks maximise search efficiency of mobile foragers. Recently, several studies raised some concerns about the reliability of the statistical analysis used in previous analyses. Further, it is unclear whether Lévy walks represent adaptive strategies or emergent properties determined by the interaction between foragers and resource distribution. Thus two fundamental questions still need to be addressed: the presence of Lévy walks in the wild and whether or not they represent a form of adaptive behaviour.

**Methodology/Principal Findings:**

We studied 235 paths of solitary and clustered (i.e. foraging in group) fallow deer (*Dama dama*), exploiting the same pasture. We used maximum likelihood estimation for discriminating between a power-tailed distribution and the exponential alternative and rank/frequency plots to discriminate between Lévy walks and composite Brownian walks. We showed that solitary deer perform Lévy searches, while clustered animals did not adopt that strategy.

**Conclusion/Significance:**

Our demonstration of the presence of Lévy walks is, at our knowledge, the first available which adopts up-to-date statistical methodologies in a terrestrial mammal. Comparing solitary and clustered deer, we concluded that the Lévy walks of solitary deer represent an adaptation maximising encounter rates with forage resources and not an epiphenomenon induced by a peculiar food distribution.

## Introduction

The level of information than large herbivores have about food distribution is limited. In temperate environments grass abundance and quality depends both on predictable (season, habitat type) and unpredictable (e.g. rainfall, presence of competitors) factors. Foraging decisions are made at a range of scales from the bite-scale to the regional scale [1]. Random movement models were showed to apply to fine-scale foraging behaviour [1], [2]. The adoption of random walks at the small spatial scale which characterises the selection of foraging stations is probably cost-effective since the food content of each single station is small. At this spatial scale it may be quite useful to adopt a search strategy which may, on average, maximises encounter rate with potential food items, once the appropriate habitat patch has been selected.

Lévy walks (LW) are scale-free random walks, the step lengths of which come from probability distributions with heavy power-law tails, such that clusters of short steps are connected by rare long steps [3]. The probability density *P*(*x*) of a step length *x*, is proportional to *x* ^−*μ*^, (1<*μ*≤3). LW with *μ* = 2 maximise search efficiency [4] under some circumstances.

LW have been reported in albatrosses, fallow deer, bumblebees [4], reindeer [5], zooplankton [6], seals [7], spider monkeys [8] and goats [9], elephants [10], but recent studies [11], [12], [13], [14] presented convincing evidence that previous enthusiastic reports should be considered with caution, because unreliable statistical methods have been used to estimate *μ*. More specifically, a first demonstration [4] of Lévy walks in fallow deer was wrong because authors had mis-interpreted data reported by [2] (as themselves have recognised [12]), considering displacement time what, actually, was foraging time. Maximum likelihood approach and model selection theory should be used to discriminate between LW and alternative search patterns. Further, Lévy patterns can emerge from the interaction between non-Lévy movement and resource distribution. LW are adaptive search strategies whose adoption should confer a fitness advantage in locating food sources or mates: «most importantly, this possibility stands in contrast to the view of Lévy motion and scale invariance as epiphenomena—or even as “emergent properties”—that arise solely via interaction with the environment» [15].

In this paper we studied random walks in fallow deer (*Dama dama*). We tested the presence of Lévy movement-length distributions for 235 paths, for both solitary (70 deer, 437 moves) and clustered (mean group size 4.9±2.8 sd) deer (165 deer, 2515 moves), showing that solitary deer actually performed Lévy walks while clustered deer did not.

We used the most recent statistical methodologies proposed in literature [11], [13], [16] to overcome the problems present in previous works. In particular, we adopted rank/frequency plotting and maximum likelihood estimation (MLE), with model selection based on the Akaike Information Criterion (AIC) [12], permitting discrimination between a power-tailed distribution and an exponential alternative. We also used rank/frequency plots to discriminate between LW and composite Brownian walks (CBW) [11], [16].

Another concerns is relative to the fact that distributions with heavy-tails can originate by behavioural heterogeneity among individuals, when the analysis is made at population level [17]. Our study design, where we study a single habitat and animals perform a specific activity for most of the time (foraging) should reduce the potential for biases derived by heterogeneity. To improve reliability, we tested group composition as a potential source of heterogeneity. One can also hypothesize that the observed move distribution is indeed a mixture of distributions, as suggested in a different context by [18], finally yielding the appearance of a fat-tail [17].

In this paper we test two hypotheses: (a) the presence of LW in the studied population of fallow deer, (b) if a Lévy distribution of movements is detected, whether or not it may represents an adaptive strategy.

## Methods

**Figure 1:**
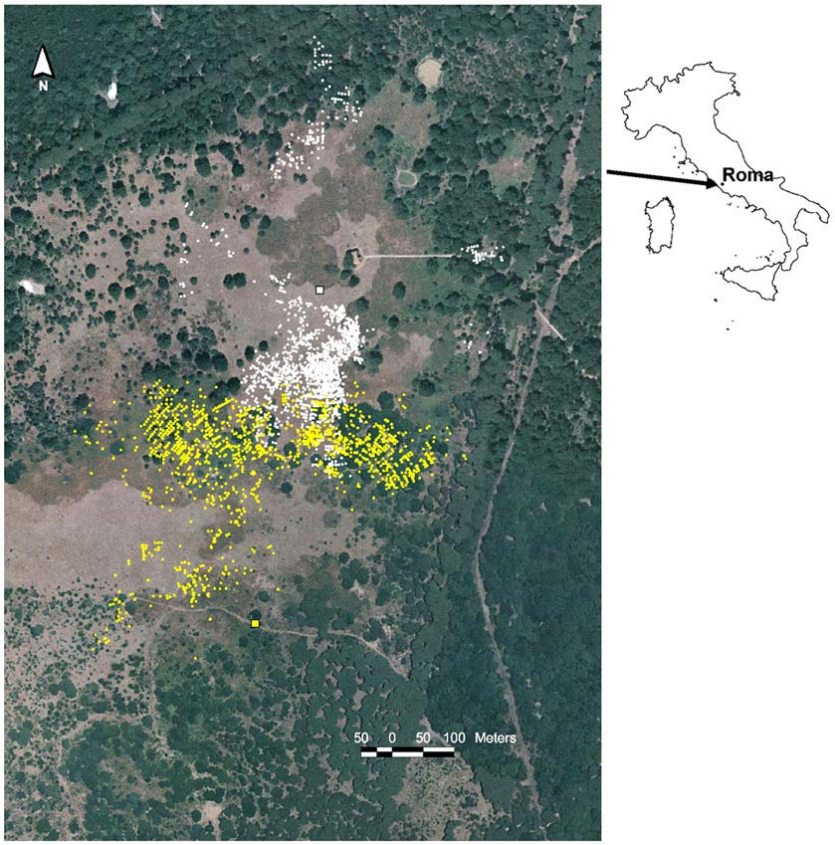
The study site. Squares denote the position of high-seats (#1 in white and #3 in yellow) while dots represent the foraging stations recorded during the study period. Food distribution is patchier in the central zone; deer foraged in meadows (light brownish open areas) or in the bushy areas near the forest's border, but did not use areas where ferns were abundant (brown open areas). No obstacle to animal movement is present in this or surrounding areas.

Behavioural observations were made in 1992 and 1993 in a pasture, surrounded by a less-productive forest (cf. [2], [19] for a detailed description of study area and field methods) at Castelporziano (Roma, Italy) (Figure 1). Fallow deer (*Dama dama*) exploit this habitat at twilight, withdrawing into the safer forest, for rest and rumination, during the daytime. Deer were observed at twilight, during peak foraging activity; the observer and the tracking device were concealed in one of 2 high-seats, 6 m above the ground, which permitted to survey a large part of the pasture (Figure 1). The tracking device consisted of an electronic compass (Ziel), and a range finder (Ranging Matic 2000) with a precision of 1 m at 150 m. In small groups, each individual could be identified by its pattern of spots and other physical features, while for large groups we used a video-camera to distinguish different animals. The sampling interval was 2–4 min (2.96±1.76 sd), depending on the difficulty of the observation. Observations could be made to a maximal distance of about 350 m. The position of each deer was determined by measuring radial distance from the observer and azimuth, then converted to Cartesian UTM co-ordinates. In this area, food distribution is uneven and food appears to be patchy distributed (Figure 1).

In order to assess if heavy-tails can originate from inter-individual heterogeneity we first looked at group composition using the following classes: adult females, yearling females, fawns (of both sexes), yearling males, and adult males. Groups were classified as small (1–3), medium (4–6) or large (>6) and we tested difference in group composition using the χ^2^ test.

Then we tested if a random effect model can improve precision in the estimate of move length in a generalised linear mixed model framework. If random effects (here group identity) would improve precision, it would be indicative of the presence of heterogeneity among groups.

In LW the step lengths come from probability distributions with heavy power-law tails [3]. This feature distinguishes a Lévy distribution from an exponential distribution where long moves are much rarer. The Lévy model specifies:
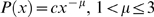
where *c* is a constant that depends on the minimal distance ecorded ([13] contains an useful discussion of the subtle implications of this model's formulation). Note that for 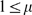, animals perform ballistic movements, and that for 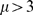 the distribution is no longer power-tailed.

The problem is how to estimate *μ*. [13] advocated the use of maximum likelihood estimation. We contrasted power-tailed and exponential models, using the AIC for model selection. Note that the observed differences of AIC values were so large that it did not deem necessary to compute Akaike weights. This analysis is appropriate whether one can prove that power-law is not generated by a mixture of exponential distribution. To test this assumption we adopted a Bayesian approach [18]. For both solitary and social deer we contrasted a single exponential distribution with a mixture of two distributions with exponential parameter *b* and *d*. The weights of the two distribution are π and (1-π). We used the inverse γ distribution as prior for *b* and *d* and the uniform distribution for π. Posterior estimates (given the observed distribution of moves) were obtained via Monte Carlo Markov Chains (MCMC). If π results to be close to 1 (or 0) we can reject the hypothesis of a mixture of distributions. According to [13], the statistical power of our samples would yield quite precise and accurate estimates using MLE (cf. [13]'s figure 3d). According to [16], we used rank/frequency plots for data display. A rank/frequency plot represents the cumulative frequency of lengths≥than any given threshold *x*. An estimate of *μ* can also be obtained from a rank/frequency plot, computing the regression coefficient, *a*, between log_10_(rank) and the log_10_(distance), where *a = μ*−1 [16]. With our samples rank/frequency estimates might be biased downward, but conserving a good precision (cf., [13]'s figure 3c).

**Figure 2:**
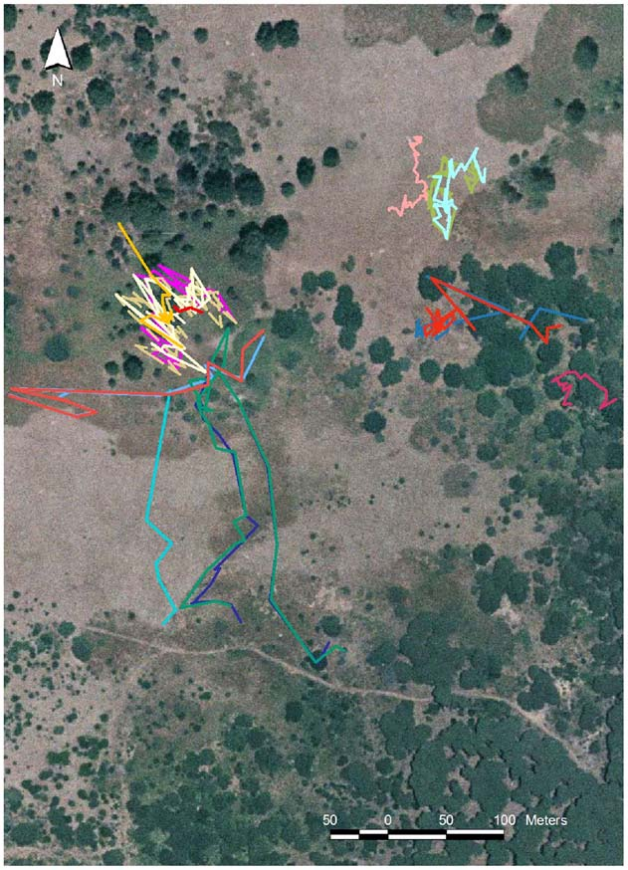
Examples of animal paths. A close-up of the study site (cf. Figure 1) shows the movement of animals in groups of different size. On the left we observed (17 June 1992, from 6:02 to 8:11) a group of seven adult females moving very sinuously and a single deer (light blue) which leaves the group and moves alone southward in a more linear pattern, albeit it stops to forage in several locations. A group composed by an adult female with its fawn (violet and green, respectively) moved on the 26 June 1992 (6:31–7:26) from the road on the south and reached the bushy area at the centre for then returning back using a different path across the pasture. In the upper zone we may observe a single animal (pink, yearling male, observed on the 29 June 1992, 19:01–20:53), moving in the same area of a group of two deer (light green and light blue, an adult female with its fawn, on the 9 September 1992, 17:05–19:33) which exhibit sinuous paths. Two adult females (blue and red) were observed on the 3 May 1992 (5:36–7:55) moving from the central forested zone to the western border. Two other adult females (blue and orange) moved eastward in the bushy area on the 31 July 1992 (18:58–20:04). A solitary female (red) moved on the 18 June 1992 (6:50–7:26) near the eastern border of the study zone.

For discriminating between LW and classical Brownian motion, we followed [11] and used a step length rank/frequency distribution (referred to as “survival distribution” by this author). In fact, LW may resemble the pattern expected for an animal which searches for patchily distributed resources by performing a composite Brownian walk, where intensive area-restricted search within patches alternates with extensive search of new patches. Thus, a composite Brownian walk (CBW) can give the illusion of a LW. [11] showed that a true LW is characterised by a linear rank/frequency plot, while a CBW shows a curvilinear plot. To discriminate between linear and curvilinear rank/frequency functions, we used a scatter plot of the residuals versus the dependent variable. Lacking any correlation between the two variables, we can conclude that the original function was linear. A trend in residuals is instead indicative of a non-linear pattern.

In interpreting the results presented here, it is appropriate to exercise some caution. A main problem, stated by [11] is related to the approximation introduced by discretization of the animal path. This problem is difficult to solve, because we can neither record the whole trajectory, nor really know at which points the animal “takes the decision” and changes direction of movement. In our case, most “animal fixes” were recorded during foraging or when the deer was vigilant, i.e., in conditions when a decision is most likely to be taken. An useful discussion about path discretization is given by [9]. On the other hand, [20] showed that the detection of LW is robust with respect to discretization. It should be noted that the treatment of data, and the highly standardised procedures for data collection ensure that the comparison between solitary and clustered deer remain valid, albeit the presence of previously-discussed limitations.

Statistical analyses were performed in SAS 9.2 (Sas Institute Inc., USA).

## Results

Some examples of trajectories are reported in Figure 2. One can note the level of synchrony in the movement of clustered deer. For instance on the left side there is a solitary deer leaving the zone with ferns and moving in a large open meadow, while a group of seven females use an area rich in bushes (greenish color) with several scattered large oaks. As well, on the upper right side of the image a single animal and a small group, formed by an adult female with its fawn, use a mixed habitat where patches of ferns (dark brown) are intermingled with, more profitable, open meadows (light brown). Some groups could be observed while foraging below the large oaks scattered in the landscape (on the right). To note that groups of every size could be observed in any part of the study area.

**Figure 3:**
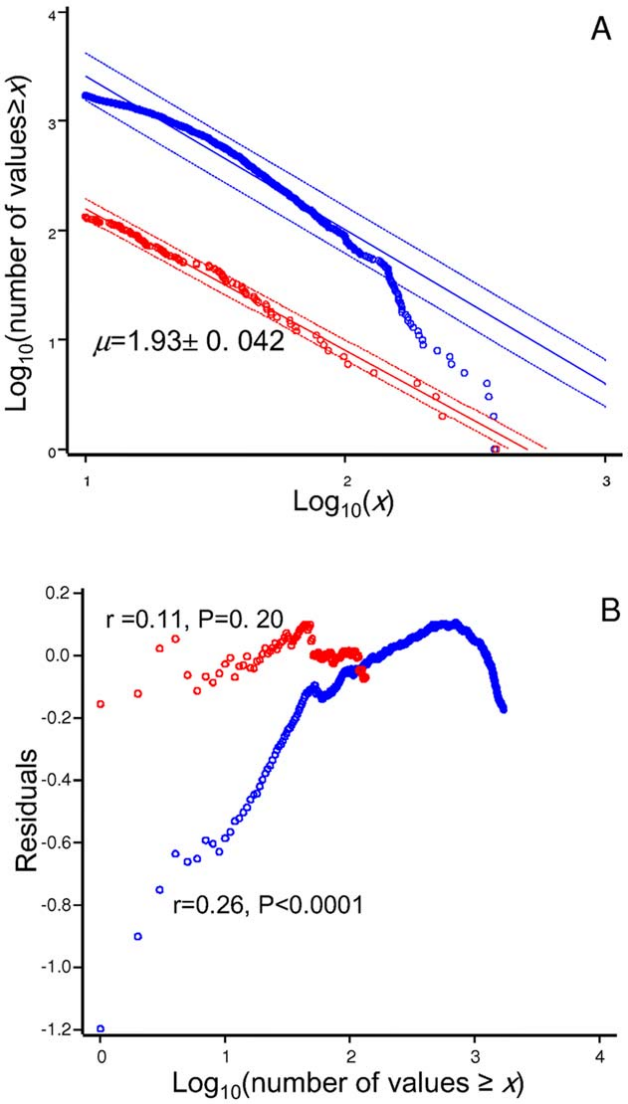
(A) Rank/frequency plots for solitary (red) and clustered (blue) deer. Only moves longer than 10 m were considered. Regressions (continuous lines) are reported with 95% confidence intervals (broken lines) for individual predicted values. Rank/frequency plots are useful to discriminate between composite Brownian walks (curvilinear) and Levy walks (linear) and are given by cumulative number of step length equal or greater than any given *x* value. In (B) we report the plots of the residual of the functions plotted in Figure 3A, as a function of the log-rank for solitary deer (red) and clustered animals (blue). The correlation between these two variables is displayed on the graph.

Group composition was quite homogenous being mainly formed by females (66.7%) and yearlings (27.6%) while the presence of yearling (3.9%) and adult males (1.9%) was occasional. No systematic difference in composition among tested groups was observed both considering all age classes (χ^2^_3_ = 4.1, P = 0.25) or males and females (χ^2^_3_ = 0.17, P = 0.91) only.

For both solitary (π = 0.90, c.i. 0.79–0.97) and social (π = 0.99, c.i. 0.98–0.99) foragers we reject the presence of a mixture of exponential distributions. For social deer we obtained a MCMC estimate equal to 25.2±0.66 m. To investigate further if group identity is relevant we estimated the exponential parameter using a random framework. We obtained a quite similar estimate (24.79 m) but a standard error (1.19) twice larger, indicating the absence of between-group heterogeneity. MLE showed that the power-tail distribution fits data better than an exponential distribution for solitary foragers (AIC_pow_ = 670.6, AIC_exp_ = 715.2), but the reverse holds for clustered animals (AIC_pow_ = 8915.0, AIC_exp_ = 8785.6). Solitary deer were characterised by a Lévy exponent (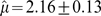) close to the optimal *μ* = 2 value, while for clustered animals the exponential parameter λ was 0.042.

The rank/frequency plot for solitary deer was quite linear (Figure 3A). The Lévy exponent was— as expected —biased low 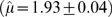 with respect to MLE (Student's test, *t*_164_ = 15.7, *P*<0.0001). Clearly, for clustered deer, the pattern was non-linear. The apparent difference among the graphs relative to solitary and clustered deer was confirmed by the distribution of residuals (Figure 3B). For solitary deer we do not observe any trend, while a significant trend is quite evident for clustered deer.

Movement patterns for solitary and clustered deer were clearly divergent during foraging. Move distribution of solitary animals was characterised by a heavy tail, i.e. long moves were substantially less frequent in clustered animals, prone to shorter displacements, strongly suggesting the adoption of CBW.

## Discussion

We confirm the presence of LW, but only in solitary deer: model selection shows that the Lévy model is well supported and the rank/frequency plot exhibits a good fitting to a Lévy distribution. This is one of the first demonstration of LW under natural conditions which can bear scrutiny based on recent methodological developments [11]–[13]. Similar conclusions have also been reported for pelagic predators [21].

Recently [22] have suggested that animal search is intrinsically discontinuous and could be described by a principle of intermittent locomotion. These authors propose the existence of background reorientation mechanisms (i.e., a fractal reorientation clocks) which generates Lévy intermittence, efficiently alternating scanning and reorientation behaviour. This hypothesis cannot be tested with our data. We have used available statistical methods to evidence differences in the statistical properties of the displacements in solitary and clustered deer.

However, provided that the same behavioral mechanism for reorientation operates in both social groups, the behavioral difference between solitary and clustered animals is explicable by mechanistic considerations. The movement of clustered deer have to be synchronised in order to maintain group coherence. When herd-mates move on, an animal is obliged to follow them, while the long moves expected in a LW would result in a loss of contact with the group.

Further we could also exclude that the heavy tail of solitary deer was originated by inter-individual heterogeneity.

Our results reject the hypothesis that LW may represent emergent properties and not adaptive search strategies. Solitary deer exhibit a nearly optimal *μ* level. If LW would arise from interactions between animals' movement and food distribution, we should consistently observe similar patterns in both clustered and solitary deer, which we did not, because all these animals experienced the same food distribution which characterises the studied pasture.

A concern is represented by the presence of finite size effects which could reduce the probability of observing the long displacements which characterized LW. Finite size effects can originate by the fact that observers cannot detect the animals if they move too far from the high seats, or by biological factors since an animal is unlikely to perform long displacements when it is close to the border of its home range. Finite size effects could not eventually explain why clustered deer present an abrupt decline in the probability of performing displacements for distance larger than about 200 m since this distance is well inside the range of animal detectability from the high seats, and small if compared to a typical home range, which in our study area are larger than 2 km^2^ [23]. We have no reasons to believe that finite size effects can operate differently for solitary and cluster deer and thus the observed differences in distance distribution should reflect actual differences in behavior and not be an effect of sampling biases. In principle truncation (in this example exponential truncation) in Lévy distribution can be tested, using a model of the kind 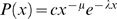. However, for statistically testing such a model it is necessary to have a very huge sample size [cf. 24], which is not our case.

It is probable that not all movements will be associated with foraging, so pooling movements related to different types of activities will complicate analysis [21]. Indeed deer during observations did not changes movement strategy by shifting among different motivational states. In this experiment deer were strongly motivated to forage and we observed them in absence of disturbances that would bias our interpretation. Basic assumptions of Lévy walks are that angles and moves are determined independently but in any field study we have to use path discretization, which could introduce a bias in the estimation of the Lévy exponent. Recently [25] gave relevant guidelines for dealing with the discretization problems (a problem raised by [11]) but they also stressed that “the hallmark power-law tail of Lévy flights is, in fact, quite robust with respect to this form of subsampling”. It is interesting to note that in a previous work of ours [2] it was showed that in this specific sample of animals there was no, or scarce, autocorrelation for both angles and distances, which strengths reliability of our results.

This study suggests a number of relevant implication for the behavioural ecology of large herbivores. The adoption of LW can be important for dispersal, when juveniles should optimise displacements through hostile habitats, without a-priori knowledge of resource distribution. Such a process would lead to population superdiffusion [15], which has clear implications for metapopulation persistence.

A pattern similar to the one we showed in fallow deer was also described in spider monkeys [8]. Noteworthy, several species of large vertebrates, reported to exhibit LW, such as albatrosses and goats were observed during solitary foraging excursions. Interestingly enough, even in the context of complex networks, the presence of social interactions in humans generates degree distributions that are not heavy-tailed [26].
